# Mitochondrial respiratory capacity is not altered in aging rat brains with or without memory impairment

**DOI:** 10.17912/micropub.biology.001359

**Published:** 2024-10-21

**Authors:** Pamela J. Yao, Jeffrey M. Long, Peter R. Rapp, Dimitrios Kapogiannis

**Affiliations:** 1 Laboratory of Clinical Investigation, National Institute on Aging Intramural Research Program, National Institutes of Health; 2 Laboratory of Behavioral Neuroscience, National Institute on Aging Intramural Research Program, National Institutes of Health

## Abstract

Mitochondria are essential for supporting the high metabolic demands that are required for brain function. Impairments in mitochondria have been linked to age-related decline in brain functions. Here, we investigate whether the mitochondrial respiratory capacity of brain cells is changed in cognitive aging. We used a rat model of normal cognitive aging and analyzed mitochondrial oxidative phosphorylation in frozen brain samples. Mitochondrial oxygen consumption rate analysis of the frontal cortex did not show any differences between young rats and aged rats with either intact memory or impaired spatial memory. Mitochondrial ATP synthase activity and quantity also did not differ between young and aged rats. These results suggest that the total level of mitochondrial respiratory capacity is preserved in the frontal cortex of aged rats and may not explain aging-associated cognitive impairment.

**Figure 1.**
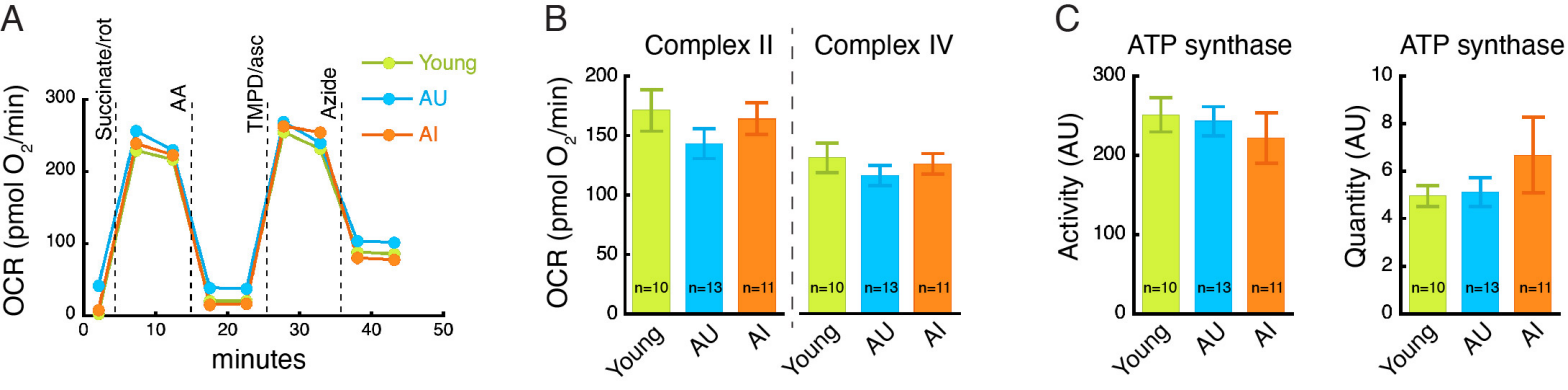
**Aged rat brains, with or without memory impairment, do not show significant changes in mitochondrial respiration capacity** . ( **A** ) Representative traces of oxygen consumption rate (OCR) of frontal cortex samples from young rats, a ged rats with u nimpaired memory (AU), and a ged rats with i mpaired spatial memory (AI). Succinate/rotenone-induced respiration represents complex II-mediated respiration; TMPD/ascorbic acid-induced respiration represents complex IV-mediated respiration. Each data point is an average of duplicate samples. ( **B** ) Quantification of (A). ( **C** ) ATP synthase specific activity (left) and quantity (right) in the frontal cortex of young and aged rats. AU, artificial unit. Data are mean ± SEM. p values were determined by unpaired Student’s *t* test. p > 0.05 for comparisons between young and AU or AI in all measurements. For (B) and (C), the number of rats used in each group is indicated on the histograms.

## Description

A decline in mitochondrial activity has been reported in relation to age-dependent functional decline in various organs (Kujoth et al., 2005; Luce and Osiewacz, 2009; Petersen et al., 2003; Rockstein and Brandt, 1963; Sun et al., 2016; Suomalainen and Nunnari, 2024) , but it is unclear how brain mitochondrial function may change with normal aging brain. In the frontal cortex of a rat model of normal cognitive aging (Cooper et al., 2024; Gallagher and Rapp, 1997) , we measured the mitochondrial oxygen consumption rate using a protocol established for frozen samples (Acin-Perez et al., 2020; Yao et al., 2023) . Figure 1A illustrates nearly identical trace profiles of oxygen consumption rate (OCR) in young rats, aged rats with unimpaired memory (AU) and aged rats with impaired memory (AI). Quantitative analysis of the respiratory chain Complex II- and Complex IV-mediated OCR did not reveal any differences between young and aged rat groups ( Fig. 1B ). The catalytic activity and quantity levels of ATP synthase (Complex V), determined by a different assay, were also not different among the rat groups ( Fig. 1C ).

We conclude that mitochondrial function, assessed at the level of mitochondrial oxidative phosphorylation activity, is preserved in the brains of aged rats with or without aging-associated memory impairment. Whether other aspects of mitochondria are changed with or contribute to age-related decline in brain functions remains to be an important topic of future research.

## Methods

*Animals and brain samples*

Young adult (6 months old) and aged (24 months old) male Long-Evans rats (Charles River Laboratories) (RRID:RGD_2308852) were used in this study. Rats were tested using a standardized water maze protocol, identical to previous descriptions (Cooper et al., 2024) , optimized for identifying individual differences in spatial memory in aging. Key features of this 8-day protocol include sparse training (3 trials/day) with probe trials interpolated over the course of training. Spatial memory in the aged rats was classified as unimpaired (AU) or impaired (AI) relative to young controls on the basis of a summary Learning Index, reflecting spatial bias for the location of the hidden escape platform during training. Animals with sensorimotor or motivational deficits detected in a visible cue platform version of the water maze were excluded from analysis. The number of rats used in this study was: young, N=10; AU, N=13; AI, N=11.

Following anesthesia and decapitation, brains were extracted and frontal cortices were dissected over ice, snap-frozen on dry ice, and stored at -80 ^0^ C. The rat sample identity was blinded to the investigator (P.J.Y) who performed the mitochondria assays until after completion of the assays.

All procedures were approved by the Animal Care and Use Committee of the Intramural Research Program of the National Institute on Aging, in accordance with the National Research Council for the Care and Use of Laboratory Animals.

*Oxygen consumption rate (OCR) measurement*

For OCR measurement, frozen brain samples were processed following a protocol optimally designed for analyzing mitochondrial oxidative function in frozen tissues (Acin-Perez et al., 2020; Yao et al., 2023) . After swiftly thawing on ice, rat frontal cortices were homogenized in cold MAS assay buffer using a pestle motor mixer (Argos) in 1.5-ml microtubes. MAS buffer: 70 mM sucrose, 220 mM mannitol, 5 mM KH _2_ PO _4_ , 5 mM MgCl _2_ , 1 mM EGTA, 2 mM HEPES, pH 7.4. The homogenates were centrifuged at 1,000 x *g* for 5 min at 4 ^o^ C, and the supernatants were collected. Protein concentration of the supernatants was determined using Pierce BCA protein assay kit (Thermo Fisher Scientific).

The samples containing 20 ug of total protein in 20 ul of the MAS buffer were loaded to each well of a Seahorse XF24 cell culture plate (Agilent). After centrifuging the plate at 2,000 x *g* for 5 min at 4 ^o^ C, 130 ul of MAS buffer containing cytochrome c (10 ug/ml) and alamethicin (10 ug/ml) was added to each well.

The sensor cartridge plate (Agilent) was loaded with substrates and inhibitors (50 ul per port) as follows: port A, succinate + rotenone (5 mM + 2 uM); port B, antimycin A (4 uM); port C, TMPD + ascorbic acid (0.5 mM + 1 mM); port D, sodium azide (50 mM). These conditions allow for the measurement of complex II- and IV-mediated maximal respiratory capacity (Acin-Perez et al., 2020; Yao et al., 2023) .

*ATP synthase (Complex V) measurement*

ATP synthase catalytic activity and quantity in the rat brain tissues were measured using a plate-based colorimetric assay (abcam; ab109716). This assay is intended for measuring both the activity and quantity of ATP synthase. The ratio of the two measurements represents the specific activity of ATP synthase.

We followed the manufacturer’s instructions with minor modifications. After thawing on ice, rat cortices were homogenized in the assay buffer (solution 1) with 10% detergent (both provided in the assay kit). The homogenates were centrifuged at 10,000 x *g* for 10 min at 4 ^o^ C, the supernatants were collected, and the protein concentration was determined using the BCA assay. The samples containing 20 ug of total protein in 50 ul of the solution 1 assay buffer were added to the wells of the assay plate. The plate was incubated overnight with gentle rocking at 4 ^o^ C followed by the measurement of ATP synthase activity and quantity per the manufacturer’s instruction.

*Data analysis*

Statistical analysis was performed using KaleidaGraph (Synergy Software). Groups were compared using unpaired Student’s *t* test. The values represent the mean ± SEM.
